# Exploring the Role of Wearable Technology in Sport Kinematics and Kinetics: A Systematic Review

**DOI:** 10.3390/s19071597

**Published:** 2019-04-02

**Authors:** Yewande Adesida, Enrica Papi, Alison H. McGregor

**Affiliations:** Department of Surgery and Cancer, Imperial College London, Charing Cross Campus, London W6 8RP, UK; e.papi@imperial.ac.uk (E.P.); a.mcgregor@imperial.ac.uk (A.H.M.)

**Keywords:** wearables, sports performance, kinematics, sensors, motion analysis, athlete, coaching

## Abstract

The aim of this review was to understand the use of wearable technology in sport in order to enhance performance and prevent injury. Understanding sports biomechanics is important for injury prevention and performance enhancement and is traditionally assessed using optical motion capture. However, such approaches are limited by capture volume restricting assessment to a laboratory environment, a factor that can be overcome by wearable technology. A systematic search was carried out across seven databases where wearable technology was employed to assess kinetic and kinematic variables in sport. Articles were excluded if they focused on sensor design and did not measure kinetic or kinematic variables or apply the technology on targeted participants. A total of 33 articles were included for full-text analysis where participants took part in a sport and performed dynamic movements relating to performance monitored by wearable technologies. Inertial measurement units, flex sensors and magnetic field and angular rate sensors were among the devices used in over 15 sports to quantify motion. Wearable technology usage is still in an exploratory phase, but there is potential for this technology to positively influence coaching practice and athletes’ technique.

## 1. Introduction

### 1.1. Background

The role of feedback in sport is of great importance, and both coaches and athletes can benefit from it as a means of improving athletic performance or minimizing injury risk. The coaching process can be highly subjective, as Jones and Wallace (2005) state: ‘Every coach or athlete brings personal interests to the coaching setting’ [1]. Ambiguity can arise in how best to develop and optimize an athlete’s performance, with the technique and approach used by the coach being reliant on their own expertise, experience and background. Rising interest and research into technology is helping to overcome this subjectivity; for example, video analysis where videos can be annotated to measure angles, allowing performance to be quantified objectively rather than be dependent on the coach’s critical eye. However, whilst such approaches provide objectivity there is a desire to provide athletes with real-time feedback.

### 1.2. Types of Systems

Motion capture systems have the ability to analyse the biomechanics of many functional and sporting tasks. Optical systems consist of cameras used to track passive or active markers placed on anatomical landmarks in order to obtain full-body capture. A systematic review by Pueo et al. (2017) stated Vicon (Oxford Metrics, Oxford, UK) and Qualisys (Qualisys AB, Göteborg, Sweden) as being the most commonly used systems in a number of different sports, from tennis to swimming to taekwondo [2]. However, due to camera set-up, these systems are limited by their capture volume, generally being confined to laboratory settings. Furthermore, the large number of markers frequently required has implications on time and can impede the performance of the tasks under investigation and, conversely, the complexity of sporting tasks frequently leads to marker occlusion. 

Wearable technology, however, is an alternative approach that has the potential to overcome these limitations. There is a range of different types of sensors, including inertial measurement units (IMUs) and microelectromechanical sensors (MEMS), containing a combination of magnetometers, accelerometers and gyroscopes. In addition, there are also flex sensors, such as those produced by Spectra Symbol (Salt Lake City, UT, USA), capable of tracking joint motion through means of changes in resistance when a force is applied to the sensor. 

A significant advantage of these wearable systems is the ability to monitor athletes in a real sport environment to provide real-time feedback, a feature not offered by video analysis. Furthermore, they are designed to be small, lightweight, wireless and unobtrusive permitting full movements while participating in a sport. This gives rise to the potential for athletes to be observed outside a laboratory setting and in their natural training environment. Sensors have been used in sports such as skiing, snowboarding and swimming that take place in extreme conditions and have the added features of being waterproof or being able to withstand cold temperatures while recording data [3,4,5,6,7,8]. However, they are not without limitations: the presence of ferromagnetic objects can distort measurements from inertial-based systems [9], and precise positioning may affect data accuracy as well as data integration introducing errors when trying to extrapolate positional data from acceleration measures [10,11]. Furthermore, using a wireless method to transfer data has the potential for loss of signal during recording time or interference from mobile phones or other devices that may be on the same transmission frequency [12]. 

### 1.3. The Adoption of Wearable Technology in Sport

A number of sports are now being seen to use wearable sensors. Monitoring player workloads in Australian football using Global Positioning System (GPS) devices has allowed energy expenditure to be analysed, a process previously done using heart rate monitors [13]. Using heart rate alone is not an accurate method of determining workload as it does not factor in speed and distance travelled during a game [13]. GPS tracking devices placed on the upper back are used to quantify the difference in the amount of work done in different player positions as well as the game intensities [13,14]. An inverse relationship was proposed by Wisbey et al. (2007) between the success of a team and the workload of the players from the use of these devices [14]. 

Wearable sensors have been used in American football to monitor concussions by measuring linear and angular head accelerations upon impact [15]. Sensors have been integrated into helmet linings and mouth guards, highlighting their unobtrusiveness, a key factor in their use in training and competition. Siegmund et al. (2015) tested two such systems, which were found to detect over 95% of impacts, providing data that would have been otherwise unobtainable [15]. Injury prevention has also been considered in baseball and volleyball where there is a problem with shoulder over-use injuries. A study performed by Rawashdeh et al. (2016) was able to classify movements of the shoulder joint, giving athletes and coaches quantifiable information [16]. 

By moving away from visual approaches, coaches are able to monitor several athletes in volleyball at once and in real-time, using the VERT inertial measurement unit (IMU) system (Version 2.0, Mayfonk Inc., Fort Lauderdale, FL, USA) to quantify jump height accurately [17] without the concern of markers being obstructed from the view of a camera. The VERT IMU is commercially available, as are other systems such as the KINEXON sensor (Kinexon Sports and Media Gmbh, Munich, Germany) used in sports such as basketball to measure player acceleration and the Nadi × yoga pants (Wearable X, New York City, NY, USA) which uses a combination of motion sensors and haptic feedback to guide yoga technique. 

### 1.4. Requirements for Wearable Technology in Sport

Optical systems are widely considered to be a gold-standard method for motion capture [18], so wearable technology should be validated against such systems and tested for reliability in order to replace them. Recommendations have been suggested by Düking et al. (2018) including confirming both inter-device and intra-device reliability, simulating the movements in the sport intended for the sensor to be used in and selecting a study population that is reflective of the ultimate intended user [19]. Concurrent criterion validity is an easy way of determining similarities or differences between the data obtained through wearable technology and a gold-standard reference. Test-retest and intra-subject reliability are important when assessing the sensor performance with relation to the participants, while sensor sensitivity is essential when considering the change of parameters with respect to time, as any sensors used need to be able to track these changes [19].

The sampling frequency of a wearable device is also important for tracking changes and is dependent on the assessed movement and variables of interest, with recommendations based on the Nyquist-Shannon sampling theorem [20]. This states that the critical sampling frequency must be a minimum of two times the highest frequency in the signal of interest to obtain all the information found in the original signal [21,22]. A consequence of having too low a sampling frequency is that relevant information can be lost.

With sensors being wireless and having their own power source that is not connected to a mains supply adds a recording lifetime to the system used. Any wearable technology needs to last at least the duration of a training or testing session or the length of a race or match. 

The increasing use of wearable sensors in sport cannot be ignored. The unobtrusive measurement systems are able to provide athletes and coaches with information regarding the range of motion, accelerations and impacts, among other indicators of performance or injury risk in real-time. The provision of objective data takes coaching to a new level, allowing more informed decision-making, yet the information collected from these sensors needs to be delivered in a format that is easy to interpret for it to be of use. 

Previous reviews have focused on inertial sensors only, such as those by Chambers et al. (2015) and Camomilla et al. (2018) despite other technologies such as pressure insoles or flex sensors which could find application in sport scenarios [23,24]. Their use is not as widespread as inertial sensors and some may require further development, but have the ability to measure biomechanical variables of use to athletes and support staff. Several reviews also exist targeting the validity, reliability and use of activity monitors to quantify energy expenditure, measure heart rate and count steps [25,26,27]. However, these measures are not able to provide indications as to how the movement was performed, thus limiting their ability to intervene to improve performance or prevent injury in a sporting population.

The aim of this study was to identify the use of wearable technology in sports as a means of measuring kinetic or kinematic variables that could be used to enhance performance or prevent injury. The focus was on sport-specific movements being performed by people who participated in these sports, while wearing a form of wearable technology. In addition to looking at the measures obtained by the devices, the collection and processing of data was also considered, as well as the lifetime of the devices and how some compared to gold standard measurements.

## 2. Materials and Methods

### 2.1. Search Strategy

The following databases were used to carry out a systematic search from inception up until 31st October 2018: Scopus, Medline, Embase, Cochrane Library, IEEE Xplore, Web of Science (Core Collection) and Engineering Village. The search terms were grouped under the following headings: ‘wearable’, ‘sensor’ and ‘sport’, with the Boolean search strategy used being ‘wearable AND sensor AND sport’. The search strategy is detailed in Table 1 and Table 2. Hand searches and screening the references of relevant articles were also performed to identify studies that may have been overlooked by the electronic searches. Retrieved articles were imported into Endnote X8 software (Clarivate Analytics, Philadelphia, PA, USA). 

### 2.2. Eligibility Criteria

Articles were included if they were: published in English; included at least one of the following outcome measures: kinematics, kinetics as obtained from wearable technology; participants took part in a sport (defined as an organized physical activity done alone or with a group); dynamic movement tasks were performed related to performance in the sport studied. Articles were excluded if they were a review or case study; were a conference abstract (except peer-reviewed abstracts); used only non-wearable devices; wearable technology was used to only quantify physical activity or spatio-temporal parameters of the sport performed; or described a potential technology not validated/used with human subjects.

### 2.3. Selection Process

Duplicates arising from searches in multiple databases were removed, and the titles and abstracts were reviewed for inclusion by two independent reviewers (Y.A. and E.P.) against the inclusion and exclusion criteria. Results from both reviewers were compared through discussion, with any conflicts being resolved by a third reviewer (A.H.M). Full texts of the remaining articles were retrieved, and these were evaluated against the inclusion criteria. 

### 2.4. Data Extraction

The details used for data extraction were modified from a review published by Papi et al. (2017) looking at the use of wearable technology to assess spinal kinematics [28]. The following details were extracted from each study: aim; sport studied; sample size; participants’ demographics (e.g., population type, age, gender, mass, height); tasks conducted; measuring system used; data acquisition/sampling; participant set-up (e.g., position of the sensors, fixation method); data processing (e.g., filter used for the signal); kinematic and kinetic variables evaluated from the sensor signals (performance indicators); statistical analysis technique; and reliability/evaluation.

### 2.5. Quality Appraisal

The review by Papi et al. (2017) was used as a basis for forming a quality assessment checklist [28]. This was based on previous reviews on motion analysis and relating to the use of technology [29,30]. 17 items were included in the checklist and each was rated between zero and two (0 = no, 1 = limited and 2 = good detail), listed in Table 3. 

## 3. Results

A total of 44,220 articles were obtained from the search, five further articles were identified from another review [23] and one from a search in Sensors. After duplicates were removed, 27,767 articles remained for title and abstract screening using the eligibility criteria set out in Section 2.2. From there, 46 full texts were assessed for eligibility. 

**Figure 1:**
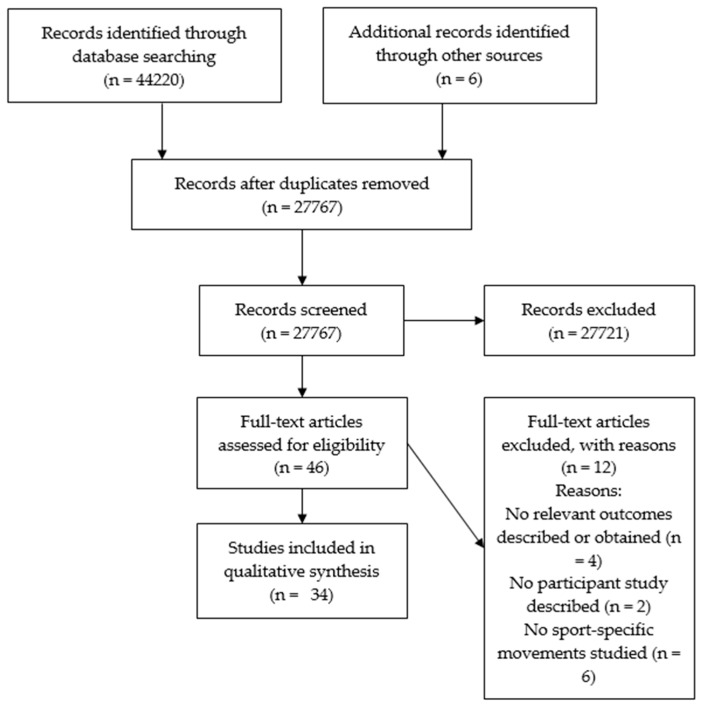
PRISMA chart detailing the article selection process [57].

Thirty-four articles satisfied the inclusion criteria. The selection process and reasons for exclusion are presented in Figure 1. The details extracted from these articles can be found in the Supplementary Materials, Table S1: Data extracted from included articles. Studies were conducted across a range of sports: football and rugby (n = 4) [9,31,32,33], swimming (n = 3) [4,6,34], skiing (n = 6) [5,7,8,35,36,37], equestrian (n = 3) [38,39,40], cricket (n = 1) [41], table tennis (n = 1) [42], badminton (n = 1) [43], athletics and running (n = 4) [12,44,45,46], rowing (n = 1) [47], baseball (n = 3) [48,49,50], snowboarding (n = 1) [3], golf (n = 1) [51], netball (n = 1) [52], archery (n = 1) [53], volleyball (n = 1) [54], canoeing (n = 1) [55] and Nordic walking [56]. 

### 3.1. Article Quality

The quality of the included papers was rated according to the following scale: low (score ≤ 33.3%), medium (33.4–66.7%) and high (score ≥ 66.8%) [28]. Four articles were deemed to be of low quality [51,53,55,56], 19 of medium quality [4,5,6,7,8,12,31,32,37,38,39,41,42,43,44,47,49,50,54] and 11 of high quality [3,9,33,34,35,36,40,45,46,48,52]. The results from this assessment are detailed in Appendix A, Table A1. None of the articles described a sampling methodology and only one article out of the 34 that were included attempted to justify the sample size [33]. Sample size was not reported in two articles [41,51] and ranged from 1 [43] to 37 [48] in the remaining articles, with the average number of participants at 10. Twelve studies had participants equal to or greater than this [4,5,7,8,9,31,33,35,36,42,48,54]. Seventeen articles described the method in enough detail to enable it to be replicated accurately [4,6,9,31,32,33,34,35,36,38,39,40,42,44,45,48,52]. All studies gave a description of where the sensors were located, with 19 of them giving a description that was clear and accurate [4,5,6,7,8,9,12,31,32,34,35,36,40,43,44,46,47,48,52]. 

Eleven of the papers compared the wearable systems to a gold-standard measurement during their testing [3,9,33,34,35,36,45,47,49,50,52]. A further four papers compared observed parameter results to values that had been reported in previous literature as means of sensor data validation [5,7,31,39].

### 3.2. Types of Measuring Systems Used and Evaluated Variables

Inertial sensors were the most common type of system used in these studies in the form of individual sensor nodes or as part of a body suit. Individual inertial sensors were used in 13 articles [6,12,32,34,38,39,45,47,48,49,52,54,55], body suits in five articles [3,6,8,9,40] and a combination in two articles [5,7].

Body suits mentioned in the included articles were produced by different companies: suits by Xsens Technologies B.V. (Enschede, The Netherlands) contained 16–17 sensor units, allowing full body coverage [3,9,37,40]; while the Physilog inertial measurement units (IMUs) (GaitUp, Lausanne, Switzerland) were incorporated into an underwear suit and contained five to seven sensors [5,7,8]. Blair et al. (2018) used the MVN Link IMS from Xsens Technologies B.V. (Enschede, The Netherlands) to determine lower body kinetics and kinematics including sagittal plane angles for the shank and pelvis during football and rugby kicks [9]; Gandy et al. (2018) also used an MVN suit to determine hip and ankle joints, as well as their moments and forces [40]; while in snowboarding the suit was used to determine knee and ankle joint angles [3]. The Physilog IMU suits (GaitUp, Lausanne, Switzerland) were all used in skiing studies by Chardonnens et al. (2013a, 2013b, 2014) measuring lower body joint angles and velocities, ski angles and centre of mass (CoM) position, force and velocity [5,7,8]. 

In archery, athletics, swimming, table tennis, baseball, football and golf, tri-axial accelerometers were used on their own to measure different kinematic and kinetic variables. Peak positive acceleration of the tibia was evaluated in runners [44]; three-dimensional acceleration and angular velocity during the golf swing using accelerometers weighing as little as 22 g [51,58]; linear and angular velocity and acceleration of the shank and thigh, as well as angular momentum, power and impulse during the football instep kick [31]. The sensor module used in football by Meamarbashi et al. had the largest dimensions of 23 cm × 2.3 cm × 2.5 cm and weighed 80 g, alongside a data logger weighing 70 g and dimensions of 6 cm × 7 cm × 2.5 cm; the placement of both components was controlled after each kick [31]. Koda et al. (2010) used the tri-axial accelerometers, weighing 93 g, to evaluate kinematics of the arm in baseball [50]. Kiernan et al. (2018) used accelerations to determine the peak vertical ground reaction force in male middle distance runners, with the accelerometer contained within an activity monitor and fixed to the lateral right iliac crest with a neoprene belt by the participants themselves [46]. In table tennis, the average peak plus acceleration value was determined with a BSN node board small enough for use on most parts of the body (23 mm in diameter) [42] and in swimming, accelerometers were used to determine roll and pitch angles (body rotation) as well as body acceleration [4]. The devices used in swimming were reported as unobtrusive and compared to every day accessories such as wristwatches and belts [4]. A tri-axial accelerometer was used alongside a load cell by Mocera et al. (2018) in Nordic walking to characterize the different phases of the cyclic arm movement [56]. The authors stated that the system must have the ‘lowest possible influence on the users’ movements in order to avoid undesirable compensations’, in a sport where the walking poles weigh as little as 180 g [56]. An accelerometer was also used in archery to measure arm displacement [53] but the type was not stated.

Magnetic, angular rate and gravity (MARG) sensors were used in football to study angle range of motion [33] and in cricket to observe the elbow extension angle [41]. The x-IMU (x-io Technologies, Bristol, UK) MARG sensors used in football weigh 49g with a battery and encased in a plastic housing (57 mm × 38 mm × 21 mm) [59]. The Pedar Pressure Insole System (novel gmbh, Munich, Germany) was used in two studies by Nakazato et al (2011, 2013) to measure the vertical ground reaction force [36] and foot centre of pressure [35] in comparison to Kistler force plates (Kistler Instruments Ltd., Hampshire, UK). The insoles have a thickness of 1.9mm and require an analyzer weighing 400 g [60]. T and T Medilogic (T and T Medilogic Medizintechnik GmbH, Schönefeld, Germany) bilateral insole measurements were used in snowboarding to determine foot force alongside an inertial suit measuring lower limb kinematics [3].

A flex sensor produced by Spectra Symbol (Salt Lake City, UT, USA) and incorporated into a glove-like hand monitoring module was used by a badminton coach in order to determine the different types of hand grips by measuring the flexion angle of the thumb, index and middle fingers [43]. In this device, the flex sensors were connected to the battery and microcontroller, which were also situated on the glove, by cables.

The specification for each technology and how each was fixed onto the study participants can be found in the Supplementary Materials (Table S1). 

### 3.3. Testing Environment

Only five studies took place in a laboratory setting [9,33,34,44,47], most likely to allow the use of a gold-standard measurement as a reference, which was the case in four articles [9,33,34,47]. Fantozzi et al. (2016) had participants simulate the upper-body portion of the swimming stroke while the lower limbs were held against a rigid box by an operator instead of performing experiments in the water [34]. Reliable measurements were produced (RMSE = 5° and 7° for breaststroke and front-crawl, respectively), but this method had its limitations as participants were required to use their lumbar muscles to support themselves and range of motion of the body was reduced in comparison to normal kinematics in the water [34]. Additionally, studies conducted by Wood et al. (2014) and King et al. (2009) used treadmills and rowing machines but both authors mentioned the need to test the wearable systems in a normal running environment and on the water [44,47].

Three articles did not state the environment that the experiments took place in [32,43,51] and the remaining studies were conducted in field, with the skiing studies by Chardonnens et al. (2013a, 2013b, 2014) and a distance running study by Kiernan et al. (2018) monitoring participants during general training [5,7,8,46]. 

### 3.4. Data Sampling, Collection and Processing

Four articles did not state the sampling frequency of the devices that were used [38,43,48,54] but for those that did, this ranged between 10 Hz and 1000 Hz, with the most common frequency used being 100 Hz by eight systems [32,35,36,37,39,49,52,55]. Sampling frequency was justified in four articles: 25 Hz was deemed ‘competent for obtaining enough knowledge of performance’ with regards to table tennis blocks by Guo et al. (2010) [42], and the accelerometers used by Kiernan et al. (2018) had a frequency that was double that of observed vertical ground reaction forces (vGRFs) in running [46]. The SportSemble nodes in baseball contained accelerometers with differing sampling frequencies (1000 Hz and 100 Hz) in order to record slow and fast motion [49]. This was recognized as a limitation of optical motion trackers. The higher sampling frequency of the SportSemble nodes enabled more detail about the pitching movement to be obtained compared to an optical motion analysis system sampling at 180 Hz, which is important when considering variables such as peak acceleration. In contrast, an inertial measurement unit (IMU) developed at Loughborough University was used by Philpott et al. (2014) to assess sprint starts in athletics had a much lower sampling frequency compared to Vicon (Oxford Metrics, Oxford, UK) (50 Hz and 250 Hz, respectively) [45]. The few data points collected by the IMU do not make it suitable for the explosive nature of the sprint start sequence. However, a sampling frequency of 50 Hz was deemed suitable for capturing the dynamics of the pole movement in Nordic walking [56]. 

Some of the systems recorded the output of the sensor onto a memory card (including micro and mini SD cards) (n = 7) [6,8,31,46,52,54,56], used data loggers (n = 3) [5,7,36] and microcontrollers (n = 3) [4,41,49]. In these cases, data was visualised and processed after testing. Some systems employed wireless data transfer (n = 11) [3,12,32,38,40,42,43,47,51,53,55], meaning that there was potential for data to be received and analysed in real-time, with five specifically stating the use of Bluetooth [38,40,43,51,53] but still data was processed after data collection.

Real-time feedback was used in only 2 articles [25,46]. Wood et al. (2014) used auditory feedback in order to reduce tibial peak-positive acceleration (PPA) in runners [44], while the “ISWIM” system used by Li et al. (2016) provided live feedback in the form of vibrations [6]. In both cases, participants were instructed to modify running or swimming biomechanics based solely on the feedback produced from the devices. Runners were able to significantly reduce PPAs throughout a 25 min testing period from 5.9 ± 0.7 g to 5.4 ± 0.7 g [44]. The case was similar with the “ISWIM” system—the vibratory feedback improved body rotation angles and as a result increased stroke rate and improved session times in three out of four participants [6]. Feedback in both systems led to a change in biomechanics without any technical input from coaches, indicating that these are systems that athletes could use on their own. 

The elbow torque-measurement device (ETD) studied by Makhni et al. (2018) used data transmission via Bluetooth to display the output of the sensor, including parameters such as arm speed, shoulder rotation and torque across the medial elbow, on a smartphone application [48]. However, the authors were not concerned with the feedback produced from the device, so there is not any indication as to its utility. This type of setup is also mentioned by Mitsui et al. (2015) to improve a golfer’s swing [51] but it was unclear whether the output was displayed in real-time or not and the impact of feedback on improving performance. 

Fifteen studies mentioned data being filtered before being subject to analysis and a variety of filters were used: second- [4,34], third- [39] and fifth- [45] order Butterworth filters; a low-pass median filter [5,7]; a second-order low-pass filter [3]; the Madgwick Altitude and Heading Reference System (AHRS) orientation filter [41,52]; a band pass filter [42]; a first-order analogue filter [49]; Kalman filtering and algorithms [9,12,34]; and a three-point moving average filter [54]. Kalman filtering was used when a wearable system contained multiple sensors in order to fuse the data, however, it is limited at higher speeds, with Blair et al. (2018) noting higher measurement errors in segments experiencing higher movement velocities in different kicking codes, but a good concurrent validity was observed when comparing the IMS to Vicon (Oxford Metrics, Oxford, UK) [9]. In three instances, commercially available data processing software from Xsens (MVN Biomech Studio, MVN Studio and MT Manager 4.2.1, Xsesns Technologies B.V.m Enschede, The Netherlands) was used to process data which uses Kalman filters to fuse sensor data [9,12,40]. In all other cases, custom-made scripts were used to process data.

### 3.5. Recording and Usage Lifetime of Wearable Systems

Systems employed different recording methods, leading to a variation in the amount of data that could be recorded and the usage lifetime. Lithium ion polymer batteries (LiPo) were found in many accelerometers, IMUs and inertial and magnetic measurement units (IMMUs) [33,49,55,61,62,63]. The exact Physilog sensors (GaitUp, Lausanne, Switzerland) used by Chardonnens et al. (2013a, 2013b, 2014) were not stated [5,7,8] but data on Physilog 4 measurement units (GaitUp, Lausanne, Switzerland) state the use of rechargeable LiPo batteries. Nickel-metal hydride (NiMH) batteries were also used, chosen for their cost-efficiency and availability [35,36,43]. The highest storage capacity listed was of the Opal IMMUs (APDM Wearable Technologies, Portland, OR, USA) which are able to store approximately 720 h of data internally used by Fantozzi et al. (2016) [34,64]. The SwimMaster system is able to record continuously for up to 48 h [4]. A summary of the battery and storage features of the devices used is listed in Table 4.

### 3.6. Validation and Reliability Using Gold-Standard Measurements

The Vicon motion capture system (Oxford Metrics, Oxford, UK) was used as a gold-standard reference for wearable systems used in football [9,33], rugby [9], baseball [50], athletics [45] and netball [52]. Philpott et al. (2014) used Kistler force platforms (Kistler Instruments Ltd., Hampshire, UK) alongside the Vicon (Oxford Metrics, Oxford, UK) camera set-up [45] when looking at sprinting in athletics. Optical based systems, such as BTS Bioengineering stereo-photogrammetric system (BTS Bioengineering Corp., Quincy, MA, USA) and XOS Technologies (Wilmington, MA, USA) optical motion analysis system), were also used as gold standards when observing movement in swimming [34], rowing [47] and baseball [49]. Kistler force plates (Kistler Instruments Ltd., Hampshire, UK) were also used in snowboarding by Krüger et al. and in skiing by Nakazato et al. (2011, 2013) [3,35,36]. The reliability of the wearable systems in comparison to the gold-standard references are detailed in Table 5. Results obtained from experimentation by Chardonnens et al. (2013a, 2014), Gandy et al. (2018), Meamarbashi et al. (2010) and Munz et al. (2013) were compared to values reported in literature [5,7,31,39], detailed in Table 6.

Accuracy of the systems used were reported using different measures in other articles. The inertial measurement units (IMU) sensors in the MVN system used by Gandy et al. (2018) were stated to have a 3-dimensional orientation accuracy within 1° [40]. Gawsalyan et al. (2017) reported a typical RMSE of around 7° for the magnetic, angular rate and gravity (MARG) sensors used in upper limb motion detection in cricket [41]. The “ISWIM” system was compared to a stopwatch, not considered a gold standard, reporting an average difference of 0.56 seconds between timings [6]. Jacob et al. (2017) reported accuracy of the flex sensors as a detection percentage, displaying a 70% detection ability in identifying five badminton grips [43]. The accuracy of the elbow torque-measurement device for monitoring baseball pitches was much higher, being able to detect 97.4% of pitches thrown by a given player [48]. A wearable sensor detecting football kicks only failed to detect six kicks out of a total of 450 [32]. The IMU for assessing skill level in volleyball had an average accuracy of 94% [54]. 

### 3.7. Application of Technology

Different applications were reported for the wearable systems and included injury prevention; quantifying skill level and expertise; improving technique; and characterizing movements. Injury prevention was the motivation behind twelve studies [3,12,33,35,36,37,40,44,46,48,49,50]. For instance, in running, high tibial peak-positive accelerations (PPAs) are linked to the risk of tibial stress fracture and as mentioned in Section 3.4, the provision of auditory feedback was able to temporarily reduce PPAs in participants [44]. By correlating the pitch of the signal output to the magnitude of PPAs, athletes were able to audibly understand the impact they were generating during each step. Fatigue is considered as another risk factor in relation to injury and the change in running mechanics was observed during a marathon [12]. Despite being ideal conditions to monitor fatigue and significant changes in mechanics, data was only collected from three participants, which is not enough to produce an analysis representative of runners in general. Yet another variable was considered as an indication of injury risk by Kiernan et al. (2018)—peak vertical ground reaction force (vGRF) was measured in injured and non-injured runners during every day training [46]. A higher peak vGRF was produced by injured runners, which is something that could be used by coaches and support staff to generate a threshold for injury potential.

Injury prevention was also considered in baseball [48,49,50], with three authors of included articles assessing different parameters. Lapinski et al. (2009) studied a combination of kinetics and kinematics of the upper body [49], Makhni et al. (2018) focused on elbow torque [48] and Koda et al. (2010) observed kinematics of the upper limb [50]. A combination of accelerometers and gyroscopes was used in all three articles. 

In other studies, authors were able to accurately measure parameters that were connected to injury-risk, such as high forces produced in middle-turns in skiing [37] and hip asymmetry in horse riders [40], suggesting their possibility of influencing rehabilitation or aiding elite coaches and athletes. However, there was no indication on the utility of the results in making biomechanical changes to reduce injuries risk. Magnetic, angular rate and gravity (MARG) sensors used by Akins et al. (2015) in football showed promise of clinical utility with regards to sagittal plane movement only [33] but the impact of these measures was not demonstrated in practice.

Wearable technology was used to quantify skill level and expertise by five authors [5,7,8,38,54]. The inertial measurement units (IMUs) used to assess the skill level of volleyball players had a 94% accuracy in determining a players’ ability. The data was also compiled into a database, increasing the impact of the sensor as this information could be used by sports scientists and professional coaches [54]. Patterson et al. (2010) also used IMUs to quantify the expertise of show jumping horse riders. The authors came to the conclusion that their hypothesis was confirmed in that novice riders were more unbalanced during jumps and differences were recorded between experienced and novice riders in the variables measured [38], but there was not any statistical analysis performed to support this or explain the significance of these differences.

Movement recognition using sensors was also explored in sports such as dressage, cricket, football, rugby, badminton, rowing, swimming and table tennis [9,32,34,39,41,42,43,47]. In dressage authors reported kinematic differences between two riders with similar levels of experience [39] and in rowing, King et al. (2009) were able to distinguish between poor and good technique but this was not quantified [39,47]. Yet in the study by Guo et al. (2010), accelerations were used to characterize the table tennis block movement as well as distinguish between different athlete levels by comparing the standard deviation in acceleration [42]. Acceleration and force values were also used to characterize Nordic walking phases by Mocera et al. (2018) [56]. The hand monitoring module (HMM) for monitoring the grip in badminton had only a 70% detection ability between the five different grips [43]. Accuracy of the wearable sensor used by Kim et al. (2016) was also reported: out of 450 kicks, only six were not detected and the sensor was also able to discriminate kicking from other motion such as walking [32]. In cricket, MARG sensors were used to analyse upper limb motion in cricketers to detect potentially illegal bowling actions. 

## 4. Discussion

The aim of this review was to determine the use and application of wearable technology in sport. From the reviewed articles it can be seen that a variety of wearable systems (including inertial sensors, pressure insoles and flex sensors) were able to measure kinetic and kinematic parameters in over 15 different sports. The common themes were injury prevention, performance assessment, movement recognition and skill level classification.

Out of these themes, injury prevention is an area with great potential when the cost of injuries and harm to athletes is considered. However, this potential has not been realised when compared to the other themes. Difficulties are presented by the many definitions of sports injury in literature and the barriers to describing their incidence: defining and gaining access to the population of interest and obtaining a suitable measure of exposure time [68]. Anecdotal experience is often the basis for preventive measures, such as strength training and stretching [69]; little of it is evidence-based as there are few randomised controlled trials reported in the literature [68,69] and this is especially the case for overuse injuries [70]. Moreover, there is a lack in the use of biomechanical measures as a means of objectively preventing injury as it is not clear which measures should be used.

However, data and technology have the potential to be used to predict injury, forming the basis for individualised programmes and allowing monitoring over a period of time, as demonstrated by Kiernan et al. (2018) who studied participants over a 60-day period [46]. To fully exploit this potential in injury prevention, however, there is still a need of identifying which biomechanical data obtained from wearable technology is the most useful as a predictor of injury.

### 4.1. Quality of Articles

The quality of the included papers varied greatly, with regards to descriptions of methods, sensor location and processing techniques. The population studied also varied, with participants ranging from an amateur to professional level. As stated by Düking et al. (2018), the study population should reflect the intended user of the wearable technology as different populations behave differently [19]. There was no standardization within each sport in terms of population size and experience. Swimming was studied by Bächlin et al. (2012) and Fantozzi et al. (2016) with 16 participants (from occasional to elite swimmers) in the former article and 8 (no experience level stated) in the latter [4,34]. Smaller sample sizes can create population bias in the results, making it difficult to trust the output.

There was also a discrepancy in detail given for the tasks conducted by study participants and the description of the location of the sensors. The amount of detail given affects the ability of a study to be accurately replicated by another person. When multiple IMUs are used, the output can be combined using a model to reconstruct human motion and trajectories, but often this is dependent on using specific anatomical landmarks, so sensor placement can affect the reliability and accuracy of the reconstruction [71].

### 4.2. Wearable Systems Used

Commercially available sensors were used in the majority of studies, the most popular being those produced by Xsens Technologies B.V. (Enschede, The Netherlands) in the form of individual sensor nodes and sensor suits in seven articles in skiing, equestrian, football, rugby, running and snowboarding [3,9,12,37,38,39,40]. A possible explanation for this could be the accuracy of these inertial sensors in comparison to a gold-standard reference. The MVN Link IMS used by Blair et al. (2018) [9] was compared to the Vicon motion capture system (Oxford Metrics, Oxford, UK) and small errors were reported (0.1 to 5.8%) between the two systems. Results from MTx inertial sensors analysing pelvis motion corresponded well with previously reported values in the literature where reflective markers and infrared cameras were used to study the same movement [39,67].

The cost of these devices is what makes them prohibitive for widespread use. The MTw Awinda 3DOF Wireless Motion Tracker (Xsens Technologies B.V., Enschede, The Netherlands) costs €400 per unit [72], while the Physilog 5 inertial measurement unit (IMU) (GaitUp, Lausanne, Switzerland) is slightly more at €499 per unit [73]. When you consider that Chardonnens et al. (2013, 2014) [5,7] used seven Physilog sensors to monitor skiing kinematics just of the lower limbs, the total cost associated with the setup rises dramatically. This limits the technology to high performance or private organizations that have a budget to spend on equipment, but this is only representative of a small section of the sporting population. 

An alternative to commercially available sensors are those that have been developed in-house, which have also been shown to be comparable to motion capture systems. ADXL193 and ADXL320 accelerometers from Analog Devices were components in the 3D sensor used by Koda et al. (2010) [50] with estimation errors of about 10% but are a fraction of the cost of the x-io Technologies IMU (Bristol, UK) used by Akins et al. (2015) [33,59,74]. The ADXL193 is being sold for £18 by one supplier [75], while the x-IMU has a cost of £309 with housing and battery [59]. Other comparable features between the two devices included sampling frequency and battery lifetimes. 

The ease of use of wearable systems must be considered. Sensors requiring complex set ups or technicians are not providing additional benefit compared to motion capture systems. For instance, the elbow torque-measurement device (ETD) studied by Makhni et al. (2018) [48] and the compression sleeve housing it were positioned by technicians and this positioning was constantly monitored. However, this process takes approximately a minute and if it is easy for coaches or other baseball players to learn and carry out it could contribute to device uptake. The fact that the activity monitor employed by Kiernan et al. (2018) [46] was placed by the participants themselves demonstrates its ease of use and indicates that small changes in positioning will not have a significant impact on the device output. The ease of using the Xsens MVN suit (Xsens Technologies B.V., Enschede, The Netherlands) was mentioned by Gandy et al. (2014) [40]. As the inertial sensors are embedded in the suit, it allowed for quicker changes between participants during the study. 

Only three studies considered the impact of the technology used on participants [48,52,56] and two did so in a quantitative manner [48,52]. In a sport such as Nordic walking where the equipment is so light (180 g per pole), any technological additions must have minimal impact in terms of weight which was considered by the authors [56]. However, no indication of the weight of the acquisition system was given nor a comparison between this setup and poles that had not been equipped with any technology. This would have provided a clear indication of its impact on the participant’s movements. An important result from the study by Shepherd et al. (2017) [52] in netball was the comparison of player performance when wearing the IMU and when not wearing it. The Pearson’s correlation coefficient, used for measuring the linear dependence between the conditions of wearing the IMU or not, was approximately equal to one [52], meaning that there was no significant impact on performance when wearing the IMU, an indicator of unobtrusiveness which could help with increasing the uptake of the device. Makhni et al. (2018) [48] followed up with participants after testing and 95% indicated that they thought it was important to monitor the stress on the arm when throwing and 73% indicated that they would alter their technique based on the results of the ETD. However, there was a significant difference between the percentage of those surveyed as to whether they would use the ETD in a practice or game setting (91% compared to 41%) [48]. Further information as to why this was the case would have been useful as for understanding what athletes expect from wearable devices if they are to use them in a competition setting. 

### 4.3. Data Collection and Processing

Motion capture systems and wearable devices are able to provide athletes and coaches with more detailed analysis of the biomechanics involved in a certain sport, enhancing the methods already employed such as video analysis. However, systems such as Vicon (Oxford Metrics, Oxford, UK) have lengthy data processing times and require familiarity with how the system works. This is being considered by researchers, who recognize the importance of making data easy to interpret and also providing simple real-time feedback to athletes. The benefits of this can be seen in the systems studied by Li et al. (2016) [6] and Wood et al. (2014) [44] where vibratory and audible feedback is provided to the athletes and has been able to change movement biomechanics by reducing tibial impact and body rotation but this type of feedback is still novel. 

Both Bächlin et al. (2012) [4] and Wang et al. (2016) [55] were aware of the importance of being able to provide real-time feedback for the SwimMaster and CanoeSense monitoring systems, respectively, and this was indicated in plans for further work, which would allow continuous monitoring and swimmers to make changes without a coach [4] or a coach to assess synchronicity between athletes and its impact on canoe propulsion [55]. 

As feedback from the “ISWIM” system was provided by the device itself and not an external source, there was not a concern for signal loss. For other wearable technology considering real-time feedback as grounds or future work, wireless data transfer was employed and some devices were affected by interference and signal loss. In the case of Reenalda et al. (2016), data was transferred wirelessly from inertial and magnetic measurement units (IMMUs) to a base station (Awinda Master, Xsens Technologies B.V., Enschede, The Netherlands)) while runners completed a marathon [12]. This base station was mounted on the handlebars of a bike that travelled alongside the athlete, with the antenna raised up to make sure that elements such as road signs and other runners did not interfere. Despite this, signal loss meant that data could only be collected for three out of the five initial participants [12]. A similar problem was encountered by Gandy et al. (2014) where wireless signals were lost at a consistent location during testing, potentially due to the presence of a radio mast [40]. Wireless data transfer has been pursued as a means of providing a less invasive system, however signal loss in outdoor environments or due to other objects causing interference limits its potential.

### 4.4. Testing Environment

Only five studies were conducted in a laboratory setting [9,33,34,44,47]. The remaining studies were conducted in a sporting environment [3,4,5,6,7,8,12,31,35,36,37,38,39,40,41,42,45,46,48,49,50,52,53,54,55] with the exception of three, where the location was not stated [32,43,51]. The conditions of testing are really important as revealed by the study conducted by Fantozzi et al. (2016) where simulated swimming altered the swimmers’ biomechanics [34]. Although this is suitable for testing the reliability of the inertial and magnetic measurement units (IMMUs), it is not necessarily suitable for considering kinematic and kinetic factors relating to injury or performance. The unpredictability of the conditions in an outdoor environment in sports such as running can influence the biomechanical motions of an athlete, therefore, it is important for wearable systems to be tested in these scenarios and allow more accurate kinetic and kinematic measures to be obtained. 

Conducting tests in a sports setting has the advantage of being able to factor in elements that may affect readings, such as drag in swimming, as well as having a better understanding of how an athlete moves in their chosen sport. Furthermore, monitoring an athlete in a training or competition environment is a good test of whether or not a system is unobtrusive as its performance is under scrutiny. Under laboratory conditions, participants may expect sensors to be uncomfortable but would generally not put up with them causing discomfort or affecting their range of motion during training or competition. 

### 4.5. Application of Wearable Technology

The potential of wearable technology is huge. Across the included articles different applications were reported: the prevention of injury; characterizing movements; analysing technique and performance; and identifying skill level. Participants from a recreational to elite level were selected in the various studies, demonstrating that these devices are not just for athletes at the top of their game but have a wider target audience, increasing the impact of wearables.

There was a crossover between themes, such as the combination of movement classification and performance in the study by Shepherd et al. (2017) in netball [52], as kinematic observations can be used to influence coaching practices to achieve a more consistent forearm angle at ball release, which would increase the likelihood of scoring during a game. Addressing more than one theme elevates the utility of a technology as it means more people can benefit from it in different ways.

The demand for wearable devices is there, especially where injury is concerned: twelve studies considered factors related to injury [3,12,33,35,36,37,40,44,46,48,49,50]. When you consider injury statistics reported by Lapinski et al. (2009) in baseball [49], where the percentage of pitchers with injuries sufficient enough to prevent them from throwing increased from 50% in 1973 [76] to over 75% in 1999 [77], there is hope that technology can reverse this trend. 

Both running and baseball injuries were each studied by three different authors, each looking at different parameters. However, in each sport, only one author was able to convey the effect of the device on study participants [44,48]. It was clear to see the influence of providing feedback in the form of audible beeps directly to athletes in the case of Wood et al. (2014) [44]. This simple method is beneficial as it allows athletes to still have an awareness of their training environment without looking at visual information in the form of figures or numbers. As discussed in Section 4.2, Makhni et al. (2018) was the closest to demonstrating buy-in from athletes in terms of using the device in training [48]. Furthermore, the elbow torque-measurement device (ETD) used was linked to a smartphone application, where quantified data may be more beneficial for coaches who can use the readouts to compare athletes. There is an advantage of smartphone applications to all users in that it is integrated into a device that is used every day, additional equipment is not needed besides the sensor. 

What this information demonstrates is that we are still at an exploratory phase of using wearable technology in sports. Despite all studies being able to measure kinematic and kinetic parameters with these devices, only a few were able to translate the output into something suitable for actual use by coaches and athletes [6,44,48,51]. This opens up the possibility to future studies to explore how to take a device from the research stage to the sporting environment by considering athlete comfort and ease of interpreting device output.

It is also evident from reviewing these articles that there is a wearable technology market for both athletes and coaches. Devices that are able to give audible and vibratory feedback are more useful for athletes who can then focus on the movements they are performing and their environment, also enabling them to make biomechanical changes without the presence of a coach. Where the device output can be displayed as readout on a smartphone or tablet is more suitable for coaches, but when developing these accompanying applications, care must be taken to only provide data that is useful and easy to interpret.

### 4.6. Review Limitations

Limitations must be considered when interpreting the findings of this review. The search was limited to seven databases, albeit integrated by reference lists and hand searches to identify other relevant papers. The results of this review are also limited by the choice of search terms and inclusion criteria—using different terms and criteria may have changed the number of articles included. However, the search terms and criteria were guided by similar reviews that have been published previously. Included articles were restricted to those published in English, posing a language bias to article selection. The quality assessment checklist was formed based on a review of wearable technology for spine movement assessment [28] as a standardized tool was not found because study quality was not reported in similar reviews. 

## 5. Conclusions

This review highlighted the increase in research surrounding wearable technology as a means to measure kinetic and kinematic parameters in sport to understand movement and differentiate between skill levels. However, it is still not at a stage where there is a good translation to general usage. 

The most common type of device used were inertial measurement units, however, authors explored stand-alone accelerometers and flex sensors also, both those commercially produced and developed in-house. Devices were developed in-house as a way to reduce their cost, which will ultimately have an impact on uptake when reaching the general market. Different applications were reported, from injury prevention to assessing performance, with the long-term vision of influencing coaching practices and athlete technique. There is potential for wearable technology to be used for long-term monitoring, especially beneficial in injury prevention as it provides coaches and athletes with the capacity to observe and analyse biomechanical risk factors over a defined exposure time, with the ability to influence injury prevention models.

A significant advantage of these devices is the ability to monitor athletes in-field instead of inside a laboratory. Laboratory testing introduces many limitations, while normal sports environments are able to provide a more accurate setting for biomechanical measurements. Furthermore, a number of studies validated the wearable technology against gold-standard reference, showing good concurrent validity. Despite the measurement errors associated with inertial measurement units, they are able to provide reliable measurements of joint kinematics and as a result, are a popular choice across different sports.

Providing real-time feedback has been shown to influence technique in swimmers and runners, but this is not yet a common feature across all sports. Wireless data transfer is a necessity but signal loss needs to be minimized in order for data to be beneficial. Any output must also be easy to interpret if it is going to be adopted by athletes and coaches who may have limited experience when analysing movement biomechanics data. Wireless data transfer has been addressed in a few studies, where data was transferred to and displayed in smartphone applications. 

There have been discrepancies in the amount of detail given in the studies carried out and the wearable sensors that were used, but it is clear that they are able to provide accurate information regarding biomechanics that can be exploited in a number of ways in sport. 

## Figures and Tables

**Table 1:** Boolean search strategy.

General	Specific	
Wearable	Portable OR worn OR cloth*3 ^1^ OR “body-mounted” OR “non-invasive” OR mobile OR wearable* OR apparel OR textile OR “electronic skin”	
	Cochrane Library MeSH terms	Wearable electronic devices (exp)
	Embase MeSH terms	Non-invasive monitoring
		Clothing
	Medline MeSH terms	Clothing
		Wearable electronic devices
Sensor	Sens*3 OR goniomet* OR acceleromet* OR monitor* OR inertia* OR gyroscope* OR device* OR magnet* OR imu OR telemet* OR pressure OR strain OR conductive OR stretch* OR flexible OR smart OR electronic*1 OR electromagnetic OR microsensor*1 OR microelectronic*1	
	Cochrane Library MeSH terms	Monitoring, ambulatory
	Embase MeSH terms	Ambulatory monitoring
		Sensor
		Devices
		Monitoring
	Medline MeSH terms	Monitoring, ambulatory
Sport	Athlete*3 OR sport* OR (List of Olympic Sports, see Table 2)	
	Cochrane Library MeSH terms	Athletes
		Sports
		Exercise
		Athletic performance
		Motor activity
	Embase MeSH terms	Athletes
		Sports
		Exercise
		Training
		Sports medicine
		Motor activity
	Medline MeSH terms	Athletic performance
		Athletes
		Sports
		Exercise
		Motor activity
		Sports medicine

^1^ The asterisk (*) after the initial letters ‘cloth’ expands the search to include all terms beginning with cloth, while the number ‘3’ limits the number of characters after ‘cloth’ of the included terms.

**Table 2:** List of Olympic sports.

Olympic Sports
archery OR run*4 ^1^ OR badminton OR basketball OR boxing OR canoe* OR cycl*4 OR bik*3 OR bicycl* OR bmx OR div*3 OR equestrian OR dressage OR fencing OR football OR soccer OR golf OR gymnastics OR handball OR hockey OR judo OR pentathlon OR row*3 OR rugby OR sail*3 OR shoot*3 OR swim*4 OR taekwondo OR tennis OR trampoline OR triathlon OR volleyball OR “water polo” OR weightlifting OR wrestling OR skiing OR biathlon OR bobsleigh OR curling OR skat*3 OR luge OR “Nordic combined” OR skeleton OR snowboard*

^1^ The asterisk (*) after the initial letters ‘run’ expands the search to include all terms beginning with run, while the number ‘4’ limits the number of characters after ‘run’ of the included terms.

**Table 3:** Criteria used for the quality assessment of included articles.

Quality Assessment Criteria
1. Were the research objectives or aims clearly stated?
2. Was the study design clearly described?
3. Was the study population adequately described?
4. Were the eligibility criteria specified?
5. Was the sampling methodology appropriately described?
6. Was the sample size used justified?
7. Did the method description enable accurate replication of the measurement procedures?
8. Was the equipment design and set up clearly described?
9. Were sensors locations accurately and clearly described?
10. Was sensor attachment method clearly described?
11. Was the signal/data handling described?
12. Were the main outcomes measured and the related calculations (if applicable) clearly described?
13. Was the system compared to an acknowledged gold standard?
14. Were measures of reliability/accuracy of the equipment used reported?
15. Were the main findings of the study stated?
16. Were the statistical tests appropriate?
17. Were limitations of the study clearly described?

**Table 4:** Battery and storage features of the wearable device systems.

Device	Battery Type	Battery Life	Storage Capacity	Application
MVN Link IMS (Xsens Technologies B.V., Enschede, The Netherlands)	One battery	Ten hours	-	Rugby and football [9]
Moven suit (Xsens Technologies B.V., Enschede, The Netherlands)	-	Approximately three hours [65]		Snowboarding [3]
Opal IMMUs (APDM Wearable Technologies, Portland, OR, USA)	-	Up to 16 h depending on whether data is logged or streamed	Internal storage of up to 8GB (approximately 720 h) [64]	Swimming [34]
Physilog 4 inertial measurement unit (IMU) (GaitUp, Lausanne, Switzerland)	LiPo battery	Up to 23 h	Internal storage of 4GB, providing 9 days of storage at 200 Hz [63]	Skiing [5,7,8]
SportSemble node	145 mAh LiPo rechargeable battery	Up to three hours	Flash memory of 116 kB (allowing each node to store around 11 seconds of data)	Baseball [49]
x-IMU magnetic, angular rate and gravity (MARG) sensors (x-io Technologies Limited, Bristol, UK)	LiPo battery (on-board charging via USB) [59]	-	-	Football [33]
G-Link-LXRS tri-axial accelerometer (LORD MicroStrain, Williston, VT, USA)	22 mAh LiPo battery (at 3.7 V)	-	2 MB [61]	Running [44]
Motus sensor (Motus Global, Rockville Centre, NY, USA)	10 mAH lithium ion battery (rapid charging using a microUSB)	Up to eight hours	Store 450+ throws	Baseball [48]
SABELSense IMU	High density LiPo battery	Approximately three hours	8 GB on a micro SD card [62]	Netball [52]
IMU nodes in CanoeSense system	1200 mAHh LiPo batteries	More than six hours	-	Canoeing [55]
Hand Monitoring Module	1500 mAh NiMh batteries (at 1.5 V per cell)	-	-	Badminton [43]
Pedar pressure insole system (novel gmbh, Munich, Germany)	NiMh batteries	-	2 GB SD card [60]	Skiing [35,36]
TSND121 wearable sensors (ATR-Promotion, Kyoto, Japan)	-	Approximately six hours	5.8 h of memory storage at 100 Hz [58]	Golf [51]
SwimMaster System	250 mAh battery at 3.7 V)	Up to 48 h	1 GB of flash memory	Swimming [4]
BSN nodes	-	-	512 kB of flash memory	Rowing [47]
XSens MTw IMMUs (Xsens Technologies B.V., Enschede, The Netherlands)	-	Approximately three hours	-	Running [12]

**Table 5:** Wearable systems compared to a gold-standard reference.

Article and Sport	System Used	Gold Standard	Reliability
Akins et al. (2015)—Football [33]	Two x-IMU magnetic, angular rate and gravity (MARG) sensors (x-io Technologies Limited, Bristol, UK)	8 camera Vicon motion capture system (Oxford Metrics, Oxford, UK)	Concurrent criterion validity was assessed by comparing ankle plantar flexion, inversion and internal rotation angles between the MARG sensors and Vicon (Oxford Metrics, Oxford, UK). High correlation between sagittal plane data (r = 0.900 to 0.975) for all manoeuvres and RMSE was <5° for drop landing, drop jump and stop jump manoeuvres. Poor correlation between frontal plane data (r = −0.074 to 0.562) and RMSE > 3° for all manoeuvres. Poor correlation between transverse plane data and RMSE > 3° for all manoeuvres.
Blair et al. (2018)—Australian football, football and rugby [9]	MVN Link IMS—17 inertial sensors (Xsens Technologies B.V., Enschede, The Netherlands)	12 camera Vicon motion capture system (Oxford Metrics, Oxford, UK)	Trivial to small errors between the IMS and Vicon (Oxford Metrics, Oxford, UK) in all kinematic parameters (0.1 to 5.8%). Trivial to small differences were found (0.2 to 5.8%) were found between linear velocities (foot and pelvis), angular velocities (knee, shank and thigh), sagittal joint (knee and hip) and segment angle (shank and pelvis) means.
Fantozzi et al. (2016)—Swimming [34]	Seven Opal IMMU units (APDM Wearable Technologies, Portland, OR, USA)	7 camera SMART-DX 7000 stereo-photogrammetric system (BTS Bioengineering Corp., Quincy, MA, USA)	Better agreement between the two systems was found during breaststroke compared to front crawl (CMC = 0.99 compared to 0.97, R = 0.99 compared to 0.95 and RMSE = 5° compared to 7°).
King et al. (2009)—Rowing [47]	Three BSN nodes with inertial sensors	SMART-D system (BTS Bioengineering Corp., Quincy, MA, USA)	Mean error between the BSN nodes and BTS system: 3.6° in femur rotation, 4.0° for thoraco-lumbar rotation and 4.1° in sacrum rotation. Accuracy of BSN nodes not as fine as BTS system resolution.
Koda et al. (2010)—Baseball [50]	3D sensor containing two types of accelerometer and gyroscope	Vicon motion capture system (Vicon460, Oxford Metrics, Oxford, UK))	Correlation coefficient (R) and RMS of error calculated between estimated position by 3D sensor and position measured by the Vicon system (Oxford Metrics, Oxford, UK). For the shoulder, elbow and wrist, R in the x and y direction showed excellent agreement (>0.95) but was smaller for the z direction (0.73 to 0.92). However RMS was less than 10 cm for the z direction and between 13 cm to 18 cm for the x and y directions.
Krüger et al. (2009)—Snowboarding [3]	Moven IMS—16 sensor units (Xsens Technologies B.V., Enschede, The Netherlands); T and T Medilogic bilateral insole measurement (T and T Medilogic Medizintechnik GmbH, Schönefeld, Germany)	Three synchronized cameras; Kistler force plate (Kistler Instruments Ltd., Hampshire, UK)	The IMS system had a moderate accuracy when compared to the cameras. Mean deviation in knee angles for left leg and right leg were 4.8° and 3.1° respectively. Correlation coefficients were high (0.96 for the left knee angle and 0.77 for the right knee angle). The insoles had a milted accuracy with a mean RMSE of 28%.
Lapinski et al. (2009)—Baseball [49]	Five SportSemble nodes—inertial measurement units (IMUs)	10 camera XOS Technologies (Wilmington, MA, USA) optical motion analysis system	No statistical difference between average shoulder internal rotation velocity in pitching measured by the IMUs and XOS Technologies system was found. Average standard deviation for IMUs was 6% compared to 15% for the optical system. In batting, the average error of bat speed at time of impact was 4.8%.
Nakazato et al. (2011)—Skiing [36]	Pedar pressure insole system (novel gmbh, Munich, Germany)	Two Kistler portable force plates (Kistler Instruments Ltd., Hampshire, UK)	The mean absolute difference of the vertical ground reaction force (vGRF) mean between the two systems ranged from 0.45 to −0.23 N/BW on the outside leg, from −0.19 to −0.10 N/BW on the inside leg and from −0.25 to 0.13 N/BW during the edge changing phase. Differences were influenced by the skier’s level, skiing mode and pitch.
Nakazato et al. (2013)—kiing [35]	Pedar pressure insole system (novel gmbh, Munich, Germany)	Two Kistler portable force plates (Kistler Instruments Ltd., Hampshire, UK)	Similarity coefficients between the two systems were contrary or low in the x direction during the outside and inside phases (−0.95 to 0.26 and −0.53 to 0.40 respectively). Highly similar time characteristics were indicated in the y direction for the outside phase (0.92 to 0.96) and were lower for the inside phase (0.15 to 0.78).
Philpott et al. (2014)—Athletics [45]	Wireless IMU	14 Vicon T-Series cameras (Oxford Metrics, Oxford, UK); two Kistler force platforms (Kistler Instruments Ltd, Hampshire, UK)	The mean correlation coefficient between the IMU and Vicon (Oxford Metrics, Oxford, UK) was 0.907. The timing accuracy of the IMU was 1.26 frames and the acceleration mean accuracy was 1.81 m/s^2^.
Shepherd et al. (2017)—Netball [52]	SABELSense IMU sensor	10 camera Vicon motion capture system (Oxford Metrics, Oxford, UK)	The IMU overestimated the Vicon (Oxford Metrics, Oxford, UK) angle of the forearm at release by 4.03°, which was deemed an appropriate level of accuracy.

**Table 6:** Wearable systems compared to values reported in literature.

Article and Sport	System Used	Reliability
Chardonnens et al. (2013a)—Skiing [7]	Seven Physilog inertial measurement units (IMUs) (GaitUp, Lausanne, Switzerland)	Validity of the system was assessed by comparing ski horizontal angle at landing impact to hill slope: −0.2 ± 4.8°, max value 11.5°. When compared to literature data, differences were smaller than 6° for 75% of the angles and smaller than 15° for 90% of the angles.
Chardonnens et al. (2014)—Skiing [5]	Seven Physilog IMUs (GaitUp, Lausanne, Switzerland)	Maximum centre of mass (CoM) velocity for Group 1 was 2.51 ± 0.83 m/s and for Group 2 was 2.23 ± 0.28 m/s compared to 2.3 m/s reported in literature.
Meamarbashi et al. (2010)—Football [31]	Sensor module and data logger	Angular velocity of the shank in the x-axis of 1911.2 ± 241.6°/s is comparable with the widely accepted value reported by Nunome et al. (2006) of 2257 ± 224.6° [66]
Munz et al. (2013)—Equestrian [39]	Two MTx inertial sensors (Xsens Technologies B.V., Enschede, The Netherlands)	Inter-individual differences were found for anterior-posterior (AP) and lateral (LT) angles in sitting trot (13.3 ± 2.3° and 6.4 ± 1.1° respectively), corresponding well with values in literature (13.9 ± 2.2° and 5.1 ± 1.1° respectively) reported by Byström et al. (2009) [67]

## Supplementary Materials

The following are available online at https://www.mdpi.com/1424-8220/19/7/1597/s1, **Table S1.** Data extracted from included articles.

## Appendix A

**Table A1:** Quality assessment of included articles (L: Low, M: Medium, H: High).

**Quality Index Item Number**	**Akins et al. (2015)**	**Bächlin et al. (2012)**	**Blair et al. (2018)**	**Chardonnens et al. (2013a)**	**Chardonnens et al. (2013b)**	**Chardonnens et al. (2014)**	**Fantozzi et al. (2016)**	**Gandy et al. (2018)**	**Gawsalyan et al. (2017)**
1	2	1	2	2	2	1	2	2	1
2	2	1	2	1	1	1	2	1	0
3	2	1	2	1	2	2	2	2	1
4	2	0	0	0	0	0	0	1	0
5	2	0	0	0	0	0	0	0	0
6	2	0	0	0	0	0	0	0	0
7	2	2	2	0	0	0	2	2	0
8	1	2	2	2	2	2	2	1	1
9	1	2	2	2	2	2	2	2	1
10	1	2	2	2	2	2	2	1	0
11	0	2	1	2	1	2	2	2	1
12	2	2	2	2	2	2	2	1	1
13	2	0	2	0	0	0	2	0	0
14	2	0	2	2	0	0	2	2	2
15	2	2	2	2	2	2	2	2	1
16	2	0	2	2	2	2	2	2	1
17	2	1	2	1	2	0	2	2	2
Total score/out of 34	29	18	27	21	20	18	28	23	12
Percentage score/%	85.3	52.9	79.4	61.8	58.8	52.9	82.4	67.6	35.3
Quality category	H	M	H	M	M	M	H	H	M
**Quality Index Item Number**	**Guo et al. (2010)**	**Jacob et al. (2017)**	**Kiernan et al (2018)**	**Kim et al. (2016)**	**King et al. (2009)**	**Koda et al. (2010)**	**Krüger et al. (2009)**	**Lapinski et al. (2009)**	**Lee et al. (2017)**
1	1	2	2	1	1	2	2	2	1
2	1	0	2	0	0	2	2	1	0
3	1	1	2	1	0	2	1	1	2
4	0	0	2	0	0	0	0	0	0
5	0	0	0	0	0	0	0	0	0
6	0	0	0	0	0	0	0	0	0
7	2	1	1	2	0	1	1	1	1
8	2	2	2	2	2	2	2	1	2
9	1	2	2	2	2	1	1	1	1
10	1	2	2	1	0	2	2	2	1
11	2	0	1	0	0	0	2	2	0
12	2	2	2	2	2	2	2	2	2
13	0	0	0	0	2	2	2	2	0
14	0	1	0	1	2	2	2	2	0
15	1	1	2	2	1	2	2	1	2
16	1	0	2	0	1	2	1	0	0
17	1	1	2	1	0	0	2	2	0
Total score/out of 34	16	15	24	15	13	22	24	20	12
Percentage score/%	47.1	44.1	70.6	44.1	38.2	64.7	70.6	58.8	35.3
Quality category	M	M	H	M	M	M	H	M	M
**Quality Index Item Number**	**Li et al. (2016)**	**Makhni et al. (2018)**	**Meamarbashi et al. (2010)**	**Mitsui et al. (2015)**	**Mocera et al. (2018)**	**Munz et al. (2013)**	**Nakazato et al. (2011)**	**Nakazto et al. (2013)**	
1	1	2	2	1	1	2	2	2	
2	0	2	1	0	0	1	2	2	
3	1	2	2	0	2	2	2	1	
4	0	2	1	0	0	0	0	0	
5	0	0	0	0	0	0	0	0	
6	0	0	0	0	0	0	0	0	
7	2	2	2	0	0	2	2	2	
8	2	2	2	1	1	2	2	2	
9	2	2	2	1	1	1	2	2	
10	1	2	2	0	0	2	1	1	
11	0	0	0	0	1	2	0	0	
12	2	2	2	2	2	2	2	2	
13	0	0	0	0	0	0	2	2	
14	0	2	0	0	0	0	2	2	
15	2	2	2	1	2	2	2	2	
16	0	2	2	0	0	2	2	2	
17	0	2	0	0	1	2	2	2	
Total score/out of 34	13	26	20	6	11	22	25	24	
Percentage score/%	38.2	76.5	58.8	17.6	32.4	64.7	73.5	70.6	
Quality category	M	H	M	L	L	M	H	H	
**Quality Index Item Number**	**Patterson et al. (2010)**	**Philpott et al. (2014)**	**Reenalda et al. (2016)**	**Shepherd et al. (2017)**	**Taha et al. (2016)**	**Wang et al. (2018)**	**Wang et al. (2016)**	**Wood et al. (2014)**	
1	2	2	2	2	2	1	1	2	
2	1	1	1	1	0	1	0	2	
3	1	2	2	2	1	2	1	2	
4	0	0	0	0	0	0	0	1	
5	0	0	0	0	0	0	0	0	
6	0	0	0	0	0	0	0	0	
7	2	2	1	2	1	1	1	2	
8	2	1	2	2	1	2	2	2	
9	1	1	2	2	1	1	1	2	
10	2	0	2	2	0	0	0	0	
11	2	2	1	1	0	2	1	0	
12	2	2	2	2	2	2	2	2	
13	0	2	0	2	0	0	0	0	
14	0	2	0	2	0	2	0	0	
15	2	2	2	2	1	2	1	2	
16	0	2	2	2	0	2	0	2	
17	1	2	2	0	1	1	0	1	
Total score/out of 34	18	23	21	24	10	19	10	20	
Percentage score/%	52.9	67.6	61.8	70.6	29.4	55.9	29.4	58.8	
Quality category	M	H	M	H	L	M	L	M	
